# Core-shell Fe_3_O_4_@zeolite NaA as an Adsorbent for Cu^2+^

**DOI:** 10.3390/ma13215047

**Published:** 2020-11-10

**Authors:** Jun Cao, Peng Wang, Jie Shen, Qi Sun

**Affiliations:** 1 College of Materials and Metallurgy, Guizhou University, Guiyang 550025, China; juncaowyy@163.com (J.C.); cywandsyx@163.com (J.S.); 2 Wuhan National Laboratory for Optoelectronics, School of Optical and Electronic Information, Huazhong University of Science and Technology 1037 Luoyu Road, Wuhan 430074, China; pengwang180907@163.com

**Keywords:** Cu^2+^ adsorbent, core-shell structure, Fe_3_O_4_@zeolite NaA, chemical chelating reaction

## Abstract

Here, using Fe_3_O_4_@SiO_2_ as a precursor, a novel core-shell structure magnetic Cu^2+^ adsorbent (Fe_3_O_4_@zeolite NaA) was successfully prepared. Several methods, namely X-ray diffraction (XRD), Fourier transform infrared spectrometer (FTIR), Transmission electron microscope (TEM), Brunauer Emmett Teller (BET) and vibrating sample magnetometry (VSM) were used to characterize the adsorbent. A batch experiment was conducted to study the Cu^2+^ adsorption capacity of Fe_3_O_4_@zeolite NaA at different pH values, contact time, initial Cu^2+^ concentration and adsorbent does. It is found that the saturated adsorption capacity of Fe_3_O_4_@zeolite NaA on Cu^2+^ is 86.54 mg/g. The adsorption isotherm analysis shows that the adsorption process of Fe_3_O_4_@zeolite NaA to Cu^2+^ is more consistent with the Langmuir model, suggesting that it is a monolayer adsorption. Adsorption kinetics study found that the adsorption process of Fe_3_O_4_@zeolite NaA to Cu^2+^ follows the pseudo-second kinetics model, which means that the combination of Fe_3_O_4_@zeolite NaA and Cu^2+^ is the chemical chelating reaction. Thermodynamic analysis shows that the adsorption process of Fe_3_O_4_@zeolite NaA to Cu^2+^ is endothermic, with increasing entropy and spontaneous in nature. The above results show that Fe_3_O_4_@zeolite NaA is a promising Cu^2+^ adsorbent.

## 1. Introduction

Cu^2+^ is a bioaccumulative heavy metal pollutant resistant to degradation [1,2]. In recent years, due to the rapid development of industry, heavy metal ions represented by Cu^2+^ have been discharged into water bodies and can be biomagnified through the food chain, which poses a huge threat to human health and the ecological environment [3]. According to reports, when the Cu^2+^ concentration in the adult body exceeds 2 mg/L, it can cause various diseases, such as gastrointestinal discomfort, kidney, liver damage and even cancer [4,5]. Therefore, removing Cu^2+^ from sewage has great significance [6]. In the past years, a large number of methods have been used to remove Cu^2+^ from sewage, including extraction, chemical precipitation, and filtration [7,8,9]. Compared with these technologies, the adsorption method has obvious advantages of low cost, simple operation and high efficiency [10] and some adsorbents have been successfully developed for the adsorption of Cu^2+^ [11].

Zeolite NaA is a microporous crystalline aluminosilicate with a pore size of about 0.4 nm. Its frame structure consists of [SiO_4_]^4−^ and [AlO_4_]^5−^ tetrahedrons connected by a common oxygen atom. [12]. This special structure makes the zeolite NaA have large surface area and strong selective adsorption capacity [13]. At present, a large number of reports confirm the possibility of using zeolite NaA as an adsorbent [14,15,16]. However, the absorbed zeolite NaA is difficult to be separate from the liquid phase, which is due to its powder morphology. To solve this problem, one method is to synthesize magnetic zeolite NaA. Li et al. [17] successfully prepared a NaA magnetic zeolite in 2013. They found that under the action of an external magnetic field, the NaA magnetic zeolite can be easily separated from the liquid phase, showing excellent separation performance. However, most of the magnetic Fe_3_O_4_ nanoparticles in magnetic zeolite NaA are distributed on the surface of the zeolite NaA, which causes part of the zeolite NaA surface that can be used for adsorption being occupied by magnetic Fe_3_O_4_ nanoparticles, thus exerting an adverse effect on the adsorption performance of magnetic zeolite NaA [18]. Many new material properties will appear in materials with nanoscale dimensions, such as excellent optical, electrical, magnetic, thermal and mechanical properties. In recent years, the emergence of core-shell nanomaterials represented by Fe_3_O_4_@SiO_2_ has attracted widespread attention and has been applied to control oral insulin delivery, curcumin release, microwave absorption, photocatalytic activity and other fields [19,20,21,22,23]. It is well known that SiO_2_ in Fe_3_O_4_@SiO_2_ can be used as a silicon source to generate zeolite NaA. Moreover, when Fe_3_O_4_@SiO_2_ is used as a precursor for further reactions, the Fe_3_O_4_ contained in Fe_3_O_4_@SiO_2_ can provide products with excellent magnetic properties. The core-shell Fe_3_O_4_@zeolite NaA may be formed, which Fe_3_O_4_@SiO_2_ is used as the precursor and after adding the corresponding quality aluminum and alkali. Because the reaction to form zeolite NaA is carried out on the surface of Fe_3_O_4_@SiO_2_, the magnetic Fe_3_O_4_ nanoparticles in Fe_3_O_4_@zeolite NaA are encapsulated by zeolite NaA. In short, this novel structure retains the excellent magnetic separation performance of traditional magnetic zeolite NaA and avoids magnetic Fe_3_O_4_ nanoparticles attaching on the zeolite NaA surface, thereby solving the problem with traditional magnetic zeolite NaA as mentioned above.

**Scheme 1:**
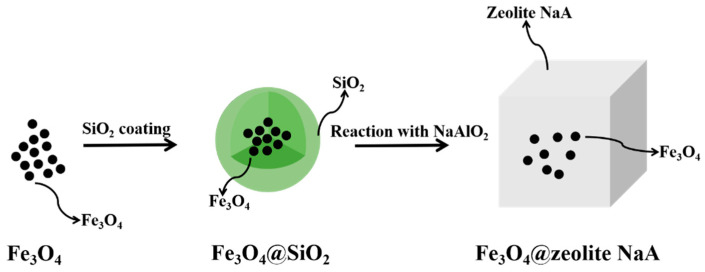
Fabrication process of Fe_3_O_4_@zeolite NaA.

In this work, a novel core-shell adsorbent Fe_3_O_4_@zeolite NaA was successfully prepared. The fabrication process of Fe_3_O_4_@zeolite NaA is shown in Scheme 1. XRD, FTIR, TEM, BET and VSM were used to characterize Fe_3_O_4_@zeolite NaA. In addition, the effect of various experimental parameters of Cu^2+^ adsorption from aqueous including pH of the solution, contact time, initial concentration and adsorbent mass, as well as adsorption kinetics, isotherm models and thermodynamics was discussed.

## 2. Experimental

### 2.1. Raw Materials

(NH_3_·H_2_O, 25%, AR), FeCl_3_·6H_2_O (AR), FeCl_2_·4H_2_O (AR), NaOH (AR), and TEOS (99.99%) were purchased from Sinopharm Chemical Reagent Co Ltd (Beijing, China). NaAlO_2_ (AR) was purchased from Shanghai Macklin Biochemical Co. Ltd (Shanghai, China). NaSiO_3_·9H_2_O (AR) was purchased from Aladdin Reagent (Shanghai, China) Co. Ltd. The deionized water was made by the laboratory.

### 2.2. Preparation of Samples

The magnetic Fe_3_O_4_ nanoparticles were prepared by a coprecipitation method. In brief, 27.03 g of FeCl_3_·6H_2_O and 9.94 g of FeCl_2_·4H_2_O (a molar ratio of 2:1) were dissolved into deionized water and mechanically stirred under a nitrogen atmosphere at 90 °C. After that, 5 mL of NH_3_·H_2_O was added dropwise into the mixture solution. The formed precipitates were collected, washed with deionized water and vacuum dried at 60 °C for 8 h.

The Fe_3_O_4_@SiO_2_ nanoparticles were synthesized by a modified method [24]. Magnetic Fe_3_O_4_ nanoparticles (20 mg) were uniformly dispersed into 20 mL ethanol under ultrasonication. Two mL of TEOS(Si(OC_2_H_5_)_4_) and 2 mL of NH_3_·H_2_O were then added under mechanical agitation to form a homogenous mixture. After that, 1:4 (V/V) deionized/ethanol solution was added dropwise under stirring within 4 h. The obtained particles were magnetically separated using a permanent magnet and washed by ethanol for several times. The samples were vacuum dried overnight at 60 °C.

The process of preparing Fe_3_O_4_@zeolite NaA is described below. The content of SiO_2_ in the prepared Fe_3_O_4_@SiO_2_ was determined by XRF analysis (Table S1). Fe_3_O_4_@SiO_2_ (20 mg) was uniformly dispersed into 2 mol/L sodium hydroxide solution under ultrasonication. 14.121 mg of NaAlO_2_ was added to the mixed solution (Si/Al ratio = 1). The reactant in the mixed solution to fully dispersed under ultrasonication. Transfer the mixed solution to the reactor and react at 110 °C for 10 h. The obtained sample was magnetically separated using a permanent magnet and washed by deionized water for several times. The sample was dried overnight at 80 °C.

The process of preparing magnetic zeolite NaA was as follows: 2 g of magnetic Fe_3_O_4_ nanoparticles, 14.21 g of NaSiO_3_·9H_2_O and 4.0985 g of NaAlO_2_ (1:1of Si/Al ratio) were mixed in 2 mol/L NaOH solution. The mixture was stirred vigorously for 2 h at 60 °C. The reactants were transferred to the reactor and crystallized for 9 h at 90 °C. The obtained sample was washed repeatedly with deionized water and dried under vacuum at 60 °C for 6 h.

### 2.3. Characterization

X-ray diffraction (XRDpatterns were recorded on a PRO XRD diffractometer (X’Pert, Holland) equipped with a Cu Kα radiation source operating at 40 kV and 100 mA. X Ray Fluorescence (XRF) were analysed on a Axios spectrometer (PANalytical, Holland). Vibrating sample magnetometry (VSM) tests were performed on a PPMS DynaCool magnetometer (Quantum Design, America). Fourier transform infrared spectra (FTIR) were obtained by the KBr wafer technique using a Ft-5dx infrared spectrometer (America) in the 4000–400 cm^−1^ region. The N^2^ adsorption isotherms of the samples were measured using N^2^ at 77 K using a 3H-2000PS4 analyzer (China). The samples were degassed at 300 °C for 5 h before the measurement (BET). The microstructure was analyzed by transmission electron microscope (TEM, Tecnai F30, Philips FEI, Holland). The structural feature and crystallinity of the samples were analyzed by an AXS D8-Focus system (Bruker Berlin, Germany). The degree of crystallinity was estimated by taking the ratio of summation of peak height of the peaks appearing at 2θ = 7.2°, 10.3°, 12.6°, 16.2°, 21.8°, 24.0°, 27.2°, 29.9° and 34.2° of the test sample to the summation of peak height of the same peaks in the commercial zeolite 4A, the crystallinity was calculated by Equation (1) [25]:

(1) $$\%\mathrm{Crystallinity}=\left(\frac{\sum^{\;}\mathrm{peak}\;\mathrm{height}\;\mathrm{of}\;\mathrm{sample}}{\sum^{\;}\mathrm{peak}\;\mathrm{height}\;\mathrm{of}\;\mathrm{commerical}\;\mathrm{zeolite}}\right)\times 100\%$$

### 2.4. Preparation of Heavy Metal Solutions

Prepared a standard Cu^2+^ stock solution (500 mg/L) by dissolving Cu(SO_4_)_2_·5H_2_O in a beaker. The solution was then diluted to the desired concentration using deionized water (see the Supporting Information S1 for detailed operational steps).

### 2.5. Adsorption Experiments

In this paper, the adsorption capacities of Fe_3_O_4_@zeolite NaA and magnetic zeolite NaA for Cu^2+^ were studied. The influence of pH, contact time, adsorbent mass and Cu^2+^ initial concentration were optimized by varying one factor while keeping others constant. Equations (S1) and (S2) were used to calculate the adsorption content of Cu^2+^ and the removal rate of Cu^2+^ by adsorbent (see the details in Supporting Information S2). Adsorption kinetics, adsorption isotherm and thermodynamic have been studied (see the details in Supporting Information S2).

## 3. Results and Discussion

### 3.1. Characterization of Samples

**Figure 1:**
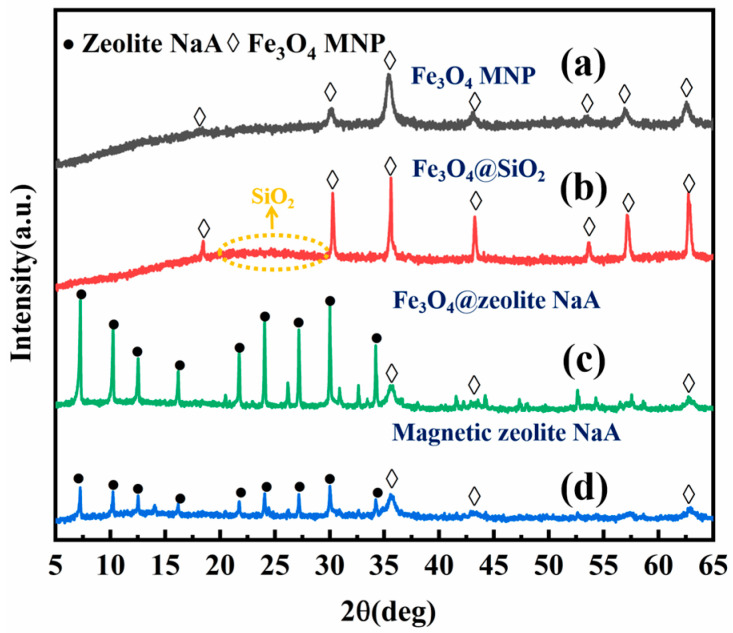
XRD patterns of (**a**) Fe_3_O_4_, (**b**) Fe_3_O_4_@SiO_2_, (**c**) Fe_3_O_4_@zeolite NaA and (**d**) Magnetic zeolite NaA.

The XRD patterns of magnetic Fe_3_O_4_ nanoparticle, Fe_3_O_4_@SiO_2_, Fe_3_O_4_@zeolite NaA and magnetic zeolite NaA are shown in Figure 1. The characteristic peaks are at 2θ = 18.3°, 30.1°, 35.5°, 43.1°, 53.5°, 57.1° and 62.6° (Figure 1a), which is characteristic of the (111), (220), (311), (400), (422), (511) and (440) crystal planes of Fe_3_O_4_ (JCPDS 88-0315), respectively, and it is in agreement with the previously reported article [26]. Compared with magnetic Fe_3_O_4_ nanoparticles, the XRD pattern of Fe_3_O_4_@SiO_2_ not only contains the characteristic magnetic Fe_3_O_4_ nanoparticle peaks, but also a slightly wider peak at 2θ = 20°–30° (Figure 1b), which corresponds to a coating of amorphous SiO_2_ [27]. In Figure 1c, the XRD pattern of Fe_3_O_4_@zeolite NaA has characteristic peaks of magnetic Fe_3_O_4_ nanoparticle and zeolite NaA. The characteristic peaks at 2θ = 35.5°, 43.1° and 62.6° are attributed to magnetic Fe_3_O_4_ nanoparticle, and at 2θ = 7.2°, 10.3°, 12.5°, 16.1°, 21.7°, 24.0°, 27.2°, 29.9° and 34.2° peaks, which are the main characteristic diffraction peaks of NaA zeolite (JCPDS 00-039-0222) and correspond to the (200), (220), (222), (420),(600), (622), (642), (644) and (664) crystal planes of NaA zeolite, respectively [28]. Except for the characteristic peaks of magnetic Fe_3_O_4_ nanoparticle and zeolite NaA, no other characteristic peaks are observed. This result indicates that no new substances are formed during the synthesis of Fe_3_O_4_@zeolite NaA. The XRD pattern of magnetic zeolite NaA (Figure 1d) shows similar characteristic peaks to Fe_3_O_4_@zeolite NaA. 

**Figure 2:**
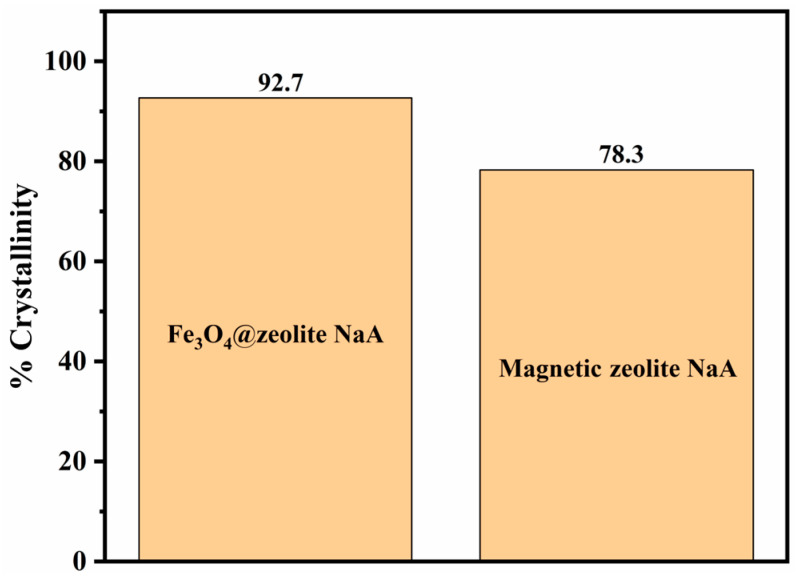
The crystallinity degree of Fe_3_O_4_@zeolite NaA and magnetic zeolite NaA.

By XRD analysis, no difference between magnetic zeolite NaA and Fe_3_O_4_@zeolite NaA can be found, so more work must be done to confirm the superiority of Fe_3_O_4_@zeolite NaA. Figure 2 shows the crystallinity of magnetic zeolite NaA and Fe_3_O_4_@zeolite NaA. It can be seen that the crystallinity of Fe_3_O_4_@zeolite NaA (92.7%) is higher than that of magnetic zeolite NaA, which means that the presence of magnetic Fe_3_O_4_ nanoparticle in Fe_3_O_4_@zeolite NaA has a smaller effect on the formation of zeolite NaA.

**Figure 3:**
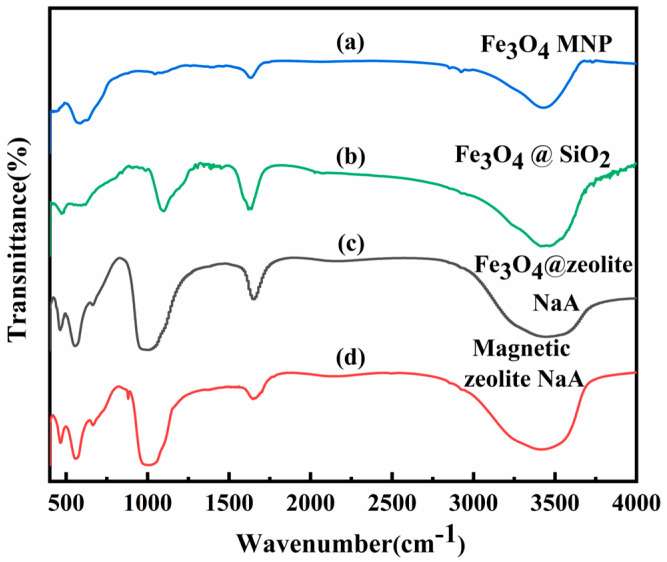
FTIR patterns of (**a**) Fe_3_O_4_, (**b**) Fe_3_O_4_@SiO_2_, (**c**) Fe_3_O_4_@zeolite NaA and (**d**) Magnetic zeolite NaA.

Figure 3 shows the FTIR spectra of magnetic Fe_3_O_4_ nanoparticle, Fe_3_O_4_@SiO_2_, Fe_3_O_4_@zeolite NaA and magnetic zeolite NaA. In the spectra of magnetic Fe_3_O_4_ nanoparticle and Fe_3_O_4_@SiO_2_ (Figure 3a,b), the peaks at approximately 620 cm^−1^, 1680 cm^−1^ and 3440 cm^−1^ correspond to Fe–O stretching vibrations [29], H–O–H bending vibrations [30] and O–H stretching vibrations [31]. In addition, in the FTIR spectrum of Fe_3_O_4_@SiO_2_, the peak at about 1100 cm^−1^ corresponds to the stretching vibration of the Si–O bond [32], proves that the sample contains SiO_2_. The FTIR spectra of Fe_3_O_4_@zeolite NaA and magnetic zeolite NaA are shown in Figure 3c,d.

It can be clearly seen that both Fe_3_O_4_@zeolite NaA and magnetic zeolite NaA have characteristic bands at 470 cm^−1^, 563 cm^−1^, 1005 cm^−1^, 1680 cm^−1^ and 3440 cm^−1^, respectively. Except for these peaks, no other peaks appear in the FTIR spectrum of Fe_3_O_4_@zeolite NaA and magnetic zeolite NaA. The peak at 470 cm^−1^ [33] is related to the internal bond vibration of the TO4 (T = Si or Al) tetrahedron in the zeolite structure. Compared with Fe_3_O_4_@SiO_2_, the peaks corresponding to Fe–O stretching vibrations and stretching vibration of the Si–O bond are shifted to 563 cm^−1^ and 1005 cm^−1^, respectively. The characteristic bands at 1680 cm^−1^ and 3440 cm^−1^ correspond to the H–O–H bending vibrations and O–H stretching vibrations, respectively. The FTIR spectrum can show that Fe_3_O_4_@zeolite NaA and magnetic zeolite NaA contain similar functional groups.

**Figure 4:**
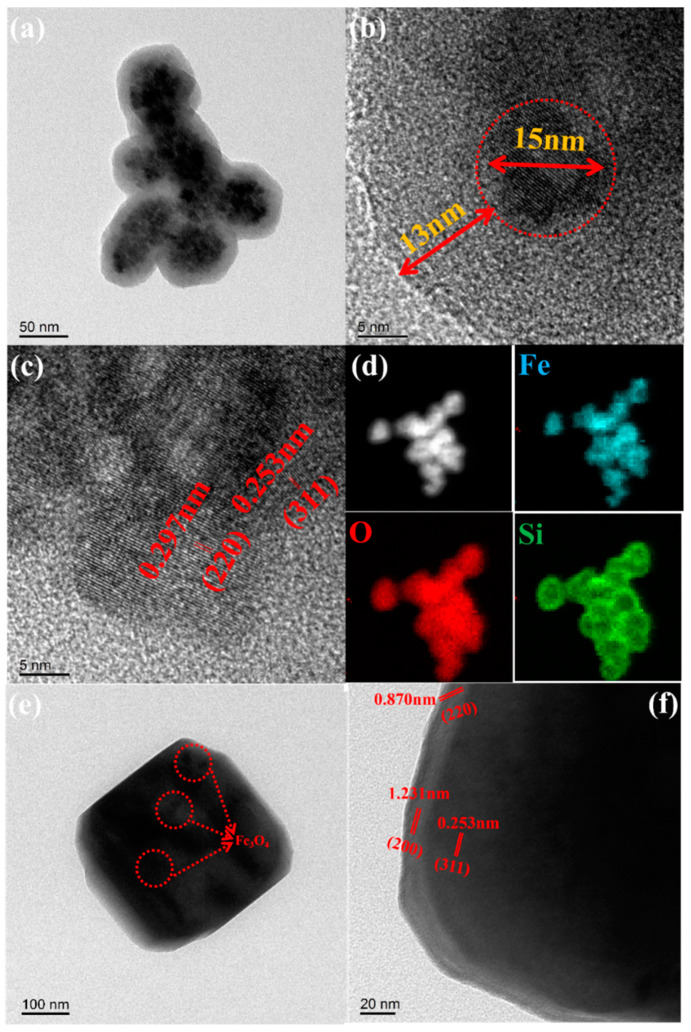
(**a**) and (**b**) TEM images of the Fe_3_O_4_@SiO_2_ (**c**) HRTEM images of Fe_3_O_4_@SiO_2_ (**d**) Element mapping of the Fe_3_O_4_@SiO_2_ based on Fe, O and Si (**e**) TEM image of the Fe_3_O_4_@zeolite NaA (**f**) HRTEM image of Fe_3_O_4_@zeolite NaA.

In order to determine the microstructure and morphology of the sample, transmission electron microscopy (TEM) is effectively used for microscopic inspection. Figure 4a shows that the well-dispersed Fe_3_O_4_@SiO_2_ nanoparticles are spherical and the surface is relatively smooth. Among them, the black center part is a magnetic core composed of multiple magnetic Fe_3_O_4_ nanoparticles. The diameter of a single magnetic Fe_3_O_4_ nanoparticle is about 15 nm, and the thickness of the SiO_2_ coating layer is about 13 nm. This information can be obtained from Figure 4b. In addition, the lattice fringes of the sample can be easily seen from Figure 4c. The measured lattice spacing of 0.253 nm and 0.297 nm correspond to the (311) and (220) crystal planes of Fe_3_O_4_, respectively. The different color areas shown in Figure 4d represent the distribution of Fe, O and Si in the real structure of Fe_3_O_4_@SiO_2_, respectively. The Fe_3_O_4_ core and the SiO_2_ shell can be clearly distinguished, which directly proves the successful preparation of the core-shell morphology Fe_3_O_4_@SiO_2_. In Figure 4e, we can clearly see that Fe_3_O_4_@zeolite NaA presents a cubic morphology, and the shell formed by zeolite NaA wraps the core formed by Fe_3_O_4_. Compared with magnetic zeolite NaA (Figure S1), due to there is no magnetic Fe_3_O_4_ nanoparticle attached to the surface, it can be seen that the surface of Fe_3_O_4_@zeolite NaA is smoother. The high-resolution TEM image of Fe_3_O_4_@zeolite NaA in Figure 4f shows clear lattice spacing of 0.870 nm, 1.231 nm and 0.253 nm, corresponding to the (220) and (200) crystal planes of zeolite NaA and (311) crystal plane of Fe_3_O_4_, respectively. The TEM results further proved that Fe_3_O_4_@zeolite NaA with core-shell structure was successfully prepared.

**Figure 5:**
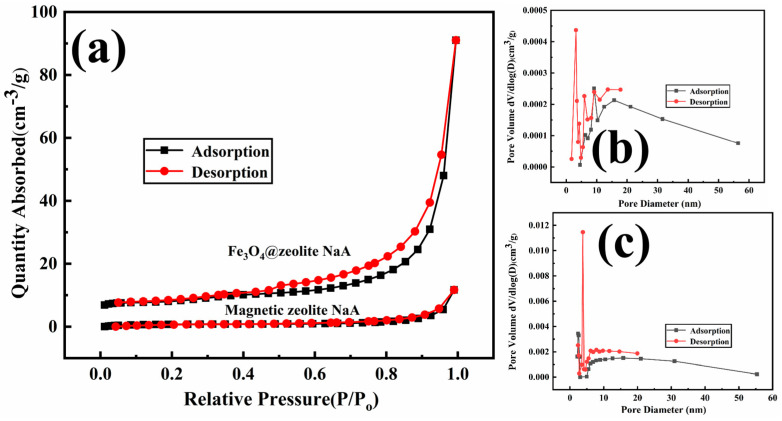
(**a**) N_2_ adsorption-desorption isotherms of Fe_3_O_4_@zeolite NaA and magnetic zeolite NaA (**b**,**c**) Pore size distributions of magnetic zeolite NaA and Fe_3_O_4_@zeolite NaA.

The specific surface area and pore structure are two important characteristics of adsorbent. The specific surface area and pore structure of magnetic zeolite NaA and Fe_3_O_4_@zeolite NaA were studied by measuring nitrogen adsorption-desorption isotherms. The results are shown in Figure 5. 

The nitrogen adsorption-desorption isotherms of both magnetic zeolite NaA and Fe_3_O_4_@zeolite NaA belong to the typical type IV isotherm [34]. Among them, the nitrogen adsorption-desorption isotherm of Fe_3_O_4_@zeolite NaA has an evident H3 hysteresis loop [35] at a relative pressure ranging from 0.5–0.9, indicating that Fe_3_O_4_@zeolite NaA contains a certain number of mesopores [36], and magnetic zeolite NaA has almost no hysteresis loop in the nitrogen adsorption-desorption isotherm. The specific surface area, average pore diameter and pore volume of the samples are shown in Table 1. The specific surface areas of magnetic zeolite NaA and Fe_3_O_4_@zeolite NaA are calculated using BET technique, which are 2.7310 m^2^/g and 26.8461 m^2^/g, respectively. It is obvious that the specific surface area of Fe_3_O_4_@zeolite NaA is much higher than that of magnetic zeolite NaA. In this study, the smaller specific surface area of magnetic zeolite NaA may be attributed to its surface being occupied by magnetic Fe_3_O_4_ nanoparticles [37], which reduces the exposed area for adsorption. Correspondingly, a large specific surface area will contain a larger number of adsorption sites [38], which will increase the possibility of adsorbed substances being removed. Figure 5b,c clearly show that magnetic zeolite NaA and Fe_3_O_4_@zeolite NaA have a wide pore size distribution. The pore volumes of magnetic zeolite NaA and Fe_3_O_4_@zeolite NaA are calculated using the BJH method, which are 0.017872 cm^3^/g and 0.136697 cm^3^/g, respectively. Obviously, the pore volume of Fe_3_O_4_@zeolite NaA is much higher than that of magnetic zeolite NaA, which means that Fe_3_O_4_@zeolite NaA has better adsorption performance [39]. The average pore diameter of Fe_3_O_4_@zeolite NaA is smaller than magnetic zeolite NaA, it may be related to the increase in the number of micropores in Fe_3_O_4_@zeolite NaA [40]. The results of nitrogen adsorption-desorption isotherms show that the specific surface area and pore volume of Fe_3_O_4_@zeolite NaA are much higher than that of magnetic zeolite NaA.

**Figure 6:**
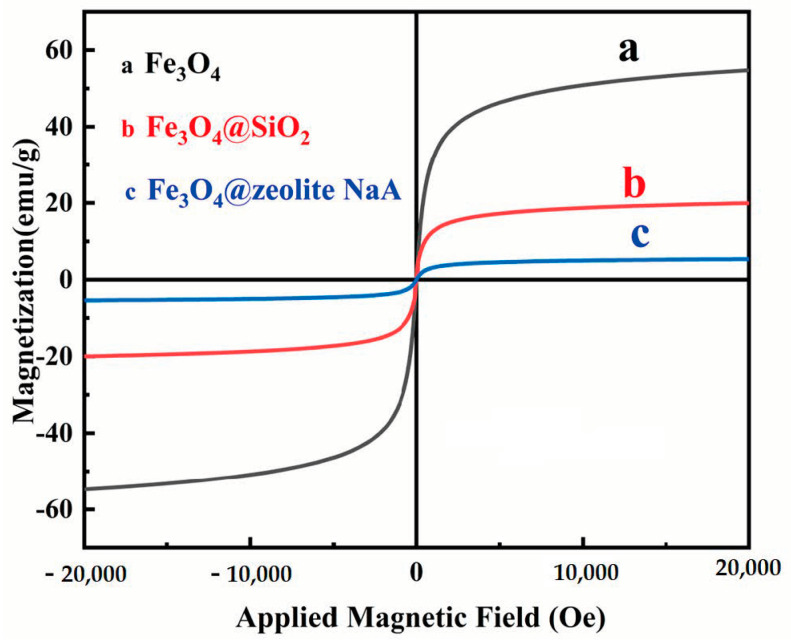
Magnetic hysteresis curves of (**a**) Fe_3_O_4_, (**b**) Fe_3_O_4_@SiO_2_ and (**c**) Fe_3_O_4_@zeolite NaA.

Figure 6 shows magnetization curves of magnetic Fe_3_O_4_ nanoparticle, Fe_3_O_4_@SiO_2_ and Fe_3_O_4_@zeolite NaA. No hysteresis effect was observed on the three samples, indicating that the prepared samples are all superparamagnetic [41]. Once the external magnetic field is withdrawn, there is no residual magnetism on the samples. It is found that the saturation magnetization value of the magnetic Fe_3_O_4_ nanoparticle is 54.79 emu/g. After being coated with SiO_2_, the saturation magnetization value of Fe_3_O_4_@SiO_2_ decreases to 19.95 emu/g, indicating that SiO_2_ has been successfully coated on the surface of the magnetic Fe_3_O_4_ nanoparticle. The saturation magnetization of Fe_3_O_4_@zeolite NaA is 5.38 emu/g, which is sufficient to ensure that Fe_3_O_4_@zeolite NaA can be separated quickly under an external magnetic field, as shown in Figure S2. Based on these results, it is proved that the Fe_3_O_4_@zeolite NaA prepared in this study has superparamagnetism and can be quickly separated from the liquid under the action of an external magnetic field.

### 3.2. Optimization of Adsorption Parameters

#### 3.2.1. Effect of pH

**Figure 7:**
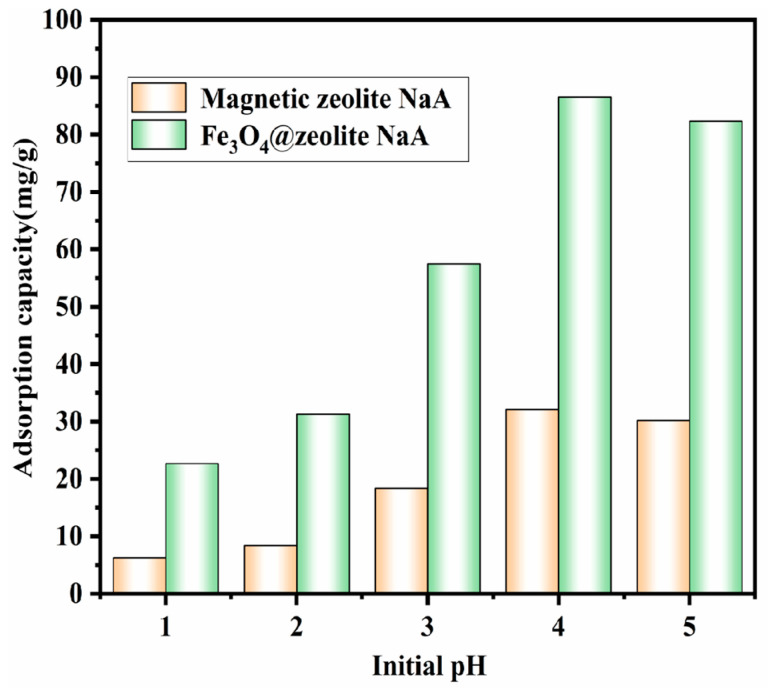
Effect of pH on the adsorption of Cu^2+^ onto magnetic zeolite NaA and Fe_3_O_4_@zeolite NaA.

The influence of various pH values (1–5) on the adsorption of Cu^2+^ by the adsorbents (2 g/L) is shown in Figure 7. We can clearly see that the adsorption capacity of Fe_3_O_4_@zeolite NaA on Cu^2+^ was enhanced when the pH increased from 1 to 4, and reaches the maximum adsorption capacity of 86.54 mg/g at pH = 4. As the pH ranged from 4.0 to 5.0, the adsorption capacity of Fe_3_O_4_@zeolite NaA on Cu^2+^ decreased significantly. When pH = 1, the low pH environment could lead to more H_3_O^+^ in the Cu^2+^ solution. These H_3_O^+^ ions compete with Cu^2+^ for active sites on the Fe_3_O_4_@zeolite NaA surface, which affects adsorption capacity of Cu^2+^ by Fe_3_O_4_@zeolite NaA. As the pH continues to increase, the amount of H_3_O^+^ continues to decrease. When pH = 2, Cu^2+^ began to become the main ion in the solution. Therefore, the adsorption capacity of Fe_3_O_4_@zeolite NaA on Cu^2+^ has experienced a substantial increase in the range of pH value is 2–4. With the increase in value of pH, the OH^-^ concentration of the solution has gradually increased and after combining with Cu^2+^, Cu(OH)_2_ begins to precipitate [42,43]. Therefore, excessive pH is not conducive to the adsorption of Cu^2+^ by Fe_3_O_4_@zeolite NaA. Under the influence of different pH values, the adsorption trend of magnetic zeolite NaA on Cu^2+^ is similar to that of Fe_3_O_4_@zeolite NaA, and reaches the maximum adsorption capacity of 32.12 mg/g at pH = 4. The result shows that at different pH, Fe_3_O_4_@zeolite NaA has a higher adsorption capacity for Cu^2+^ than magnetic zeolite NaA. Subsequent batch adsorption experiments are carried out under the condition of pH = 4. (Adsorption condition: Adsorbent dose is 0.1 g; Cu^2+^ solution volume is 50 mL; Initial Cu^2+^ concentration is 200 mg/L; Temperature is 298.15 K; Contact time is 24 min).

#### 3.2.2. Effect of Adsorbent Does on Cu^2+^ Adsorption

**Figure 8:**
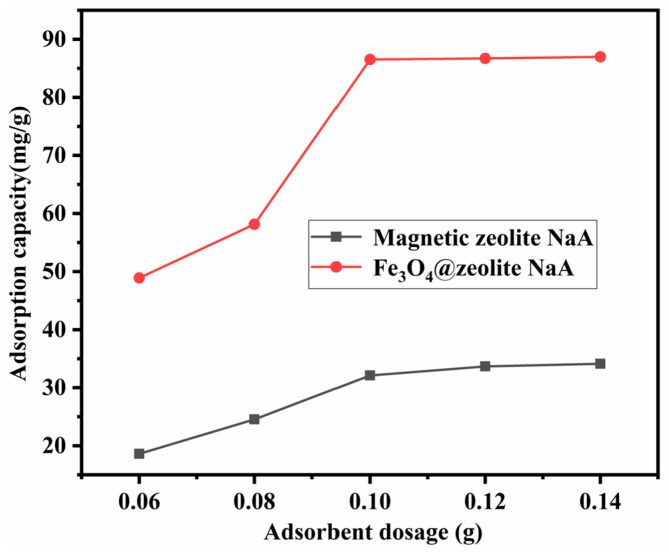
Effect of adsorbent mass on the adsorption of Cu^2+^ by magnetic zeolite NaA and Fe_3_O_4_@zeolite NaA.

As shown in Figure 8, regardless of the adsorbent used in this study (magnetic zeolite NaA or Fe_3_O_4_@zeolite NaA), the adsorption capacity of Cu^2+^ increased as the adsorbent mass increases from 0.06 g to 0.1 g, and then remained almost unchanged from 0.1 g to 0.14 g. It can be found that the maximum adsorption capacity of Cu^2+^ was found to be 86.54 mg/g for Fe_3_O_4_@zeolite NaA and 32.12 mg/g for magnetic zeolite NaA, indicating that the adsorption capacity of Fe_3_O_4_@zeolite NaA is 2.7 times that of magnetic zeolite NaA. In the subsequent adsorption experiment, the does of the adsorbent is set to 0.1 g. (adsorption condition: pH = 4; Cu^2+^ solution volume is 50 mL; Initial Cu^2+^ concentration is 200 mg/L^−1^; Temperature is 298.15 K; Contact time is 24 min)

#### 3.2.3. Effect of Contact Time on Cu^2+^ Adsorption

**Figure 9:**
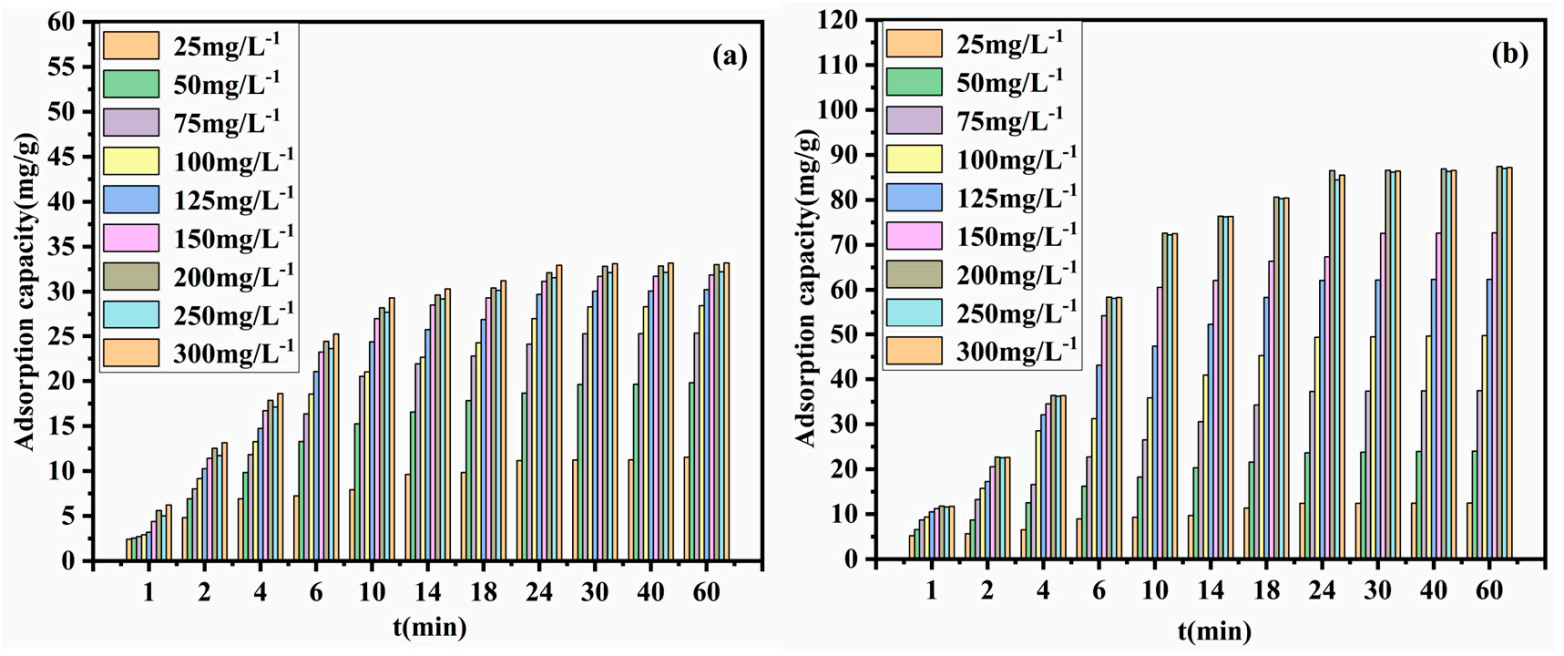
Effect of contact time on the uptake of Cu^2+^ by magnetic zeolite NaA (**a**) and Fe_3_O_4_@zeolite NaA (**b**).

At different concentrations of Cu^2+^, the effect of contact time on the removal of Cu^2+^ by magnetic zeolite NaA and Fe_3_O_4_@zeolite NaA can be seen from Figure 9a,b, respectively. 

It can be seen from the figure that when the contact time is from 1 min to 10 min, the adsorption amount of Cu^2+^ by magnetic zeolite NaA and Fe_3_O_4_@zeolite NaA increases greatly with the increase of contact time. When the contact time is from 10 min to 24 min, the adsorption amount of Cu^2+^ by magnetic zeolite NaA and Fe_3_O_4_@zeolite NaA still keeps increasing which the adsorption amount of Cu^2+^ by Fe_3_O_4_@zeolite NaA still keeps increasing greatly. When the contact time is more than the 24 min, which the adsorption capacity of magnetic zeolite NaA and Fe_3_O_4_@zeolite NaA on Cu^2+^ remained basically unchanged. It shows that the adsorption of Cu^2+^ by the adsorbent (magnetic zeolite NaA or Fe_3_O_4_@zeolite NaA) reaches the adsorption equilibrium at 24 min. The prepared Fe_3_O_4_@zeolite NaA is also compared with other adsorbents of Cu^2+^ reported in literature [44,45]. It is found that the equilibrium adsorption time of Fe_3_O_4_@zeolite NaA in this study is shorter and more suitable for practical applications. Similarly, as the concentration of Cu^2+^ increases, the amount of Cu^2+^ adsorbed by the adsorbent will also increase. The adsorption equilibrium is reached which the Cu^2+^ concentration reaches 200 mg/L. When Cu^2+^ concentration is 25 mg/L to 200 mg/L, the saturated adsorption capacity of Fe_3_O_4_@zeolite NaA on Cu^2+^ is 12.42, 23.66, 37.28, 49.36, 62.12, 67.36 and 86.54 mg/g, respectively. The saturated adsorption capacity of magnetic zeolite NaA for Cu^2+^ is 11.16, 18.66, 24.12, 27.02, 29.72, 31.16 and 32.12 mg/g, respectively. It can be seen that Fe_3_O_4_@zeolite NaA has higher adsorption capacity for Cu^2+^. In the subsequent adsorption experiment, the contact time of the adsorbent is set to 24 min. (Adsorption condition: Adsorbent dose is 0.1 g; Cu^2+^ solution volume is 50 mL; pH = 4; Temperature is 298.15 K)

#### 3.2.4. Effect of Initial Concentration

**Figure 10:**
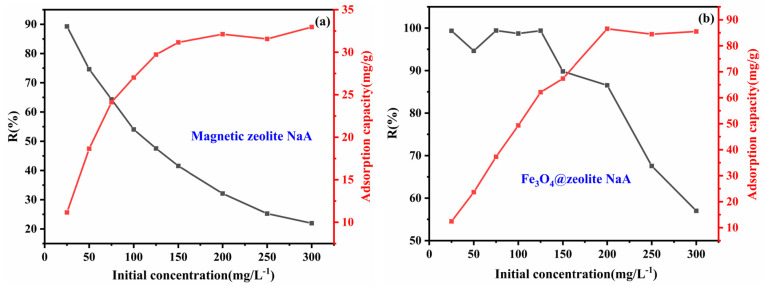
Effect of initial concentration on the adsorption of Cu^2+^ by magnetic zeolite NaA (**a**) and Fe_3_O_4_@zeolite NaA (**b**).

Figure 10 shows the effect of different initial concentrations on the adsorption performance of the adsorbents (2 g·L^−1^) for Cu^2+^. Figure 10a shows that with increase of the initial concentration, the adsorption of Cu^2+^ on magnetic zeolite NaA gradually increases. When the initial concentration reaches a certain value (200 mg/L^−1^), the adsorption capacity of magnetic zeolite NaA on Cu^2+^ reaches saturation (32.12 mg/g). It can be seen from the change curve of R_emove%_ that when the initial concentration over 100 mg/L^−1^, the R_emove%_ value of Cu^2+^ by magnetic zeolite NaA starts to be lower than 55%. When the initial concentration reaches 200 mg/L, the magnetic zeolite NaA R_emove%_ value of Cu^2+^ is only 32.12%. The above results indicate that magnetic zeolite NaA cannot be used as a Cu^2+^ adsorbent in practical applications. Figure 10b shows that the adsorption capacity of Fe_3_O_4_@zeolite NaA on Cu^2+^ increases with the increase of the initial concentration, and reaches the Cu^2+^ adsorption equilibrium when the initial concentration is 200 mg/L (the adsorption capacity is 86.54 mg/g), indicating that all adsorption sites of Fe_3_O_4_@zeolite NaA have been occupied by Cu^2+^ [46]. The change curve of R_emove%_ of Fe_3_O_4_@zeolite NaA vs.(versus) Cu^2+^ shows that as the initial concentration continues to increase, the overall trend of R_emove%_ value is downward, but the decline is small. When the initial concentration is 200 mg/L, it can still maintain 86.54%. The R_emove%_ of Cu^2+^ shows that Fe_3_O_4_@zeolite NaA can be used as an effective adsorbent to remove Cu^2+^ in sewage. The initial concentration value of the subsequent adsorption experiments are all select as 200 mg/L. (Adsorption condition: Adsorbent dose is 0.1 g; Cu^2+^ solution volume is 50 mL; pH = 4; temperature is 298.15 K; contact time is 24 min).

### 3.3. Adsorption Isotherm

**Figure 11:**
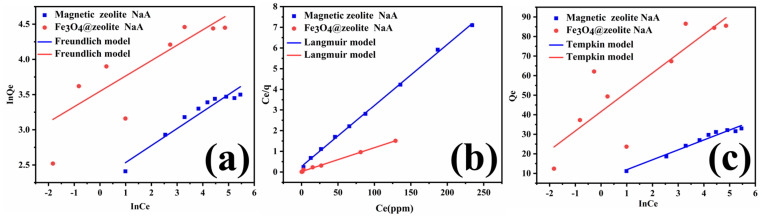
Freundlich model (**a**), Langmuir model (**b**) and Tempkin model (**c**) fittings for Cu^2+^ adsorption onto magnetic zeolite NaA and Fe_3_O_4_@zeolite NaA.

The adsorption isotherm can be used to study the binding mechanism of the adsorbents and Cu^2+^ which through the equilibrium adsorption capacity of the adsorbents to Cu^2+^ for different initial concentrations. The experimental data of adsorbent adsorption of Cu^2+^ at different initial concentrations were linearly fitted to Freundlich, Tempkin and Langmuir models, the conditions under which the pH value of 4, the adsorbent dose of 0.1 g, the Cu^2+^ solution volume of 50 mL, the contact time of 24 min and the temperature of 298.15 K. The fitting parameters of Freundlich, Tempkin and Langmuir adsorption isotherms are shown in Table 2. In Figure 11, we can clearly see that Langmuir model provides a better description of the adsorption process of magnetic zeolite NaA than Freundlich and Tempkin model. Table 2. shows that the Langmuir model of magnetic zeolite NaA has a higher R^2^ (0.99890) than the Freundlich model (0.93447) and Tempkin model (0.96782), which directly proves the above point. Combining the analysis in Figure 11 and Table 2, we found that the R^2^ value of the Freundlich model (0.61082) and Tempkin model(0.73113) of Fe_3_O_4_@zeolite NaA is smaller than Langmuir model (0.99734), which means that the Langmuir model can better describe the effect of Fe_3_O_4_ @zeolite NaA on Cu^2+^ adsorption behavior under different initial concentrations. It shows that the process of Fe_3_O_4_@zeolite NaA adsorption of Cu^2+^ is monolayer adsorption [47], the adsorption sites are uniformly distributed on the surface of Fe_3_O_4_@zeolite NaA [48]. The maximum adsorption capacity (Q_max_) of Fe_3_O_4_@zeolite NaA for Cu^2+^ calculated by the Langmuir model is 86.58 mg/g, which is very similar to the actual amount of Q_max_ (86.54 mg/g). The maximum adsorption capacity (Q_max_) of Fe_3_O_4_@zeolite NaA on Cu^2+^ is compared with other adsorbents (including magnetic zeolite NaA). The relevant results are summarized in Table 3, which proves the Fe_3_O_4_@zeolite NaA has excellent adsorption performance for Cu^2+^. As the R_L_ value lies between 0 and 1, it shows that the adsorption of Fe_3_O_4_@zeolite NaA to Cu^2+^ is spontaneous [49].

### 3.4. Adsorption Kinetics

**Figure 12:**
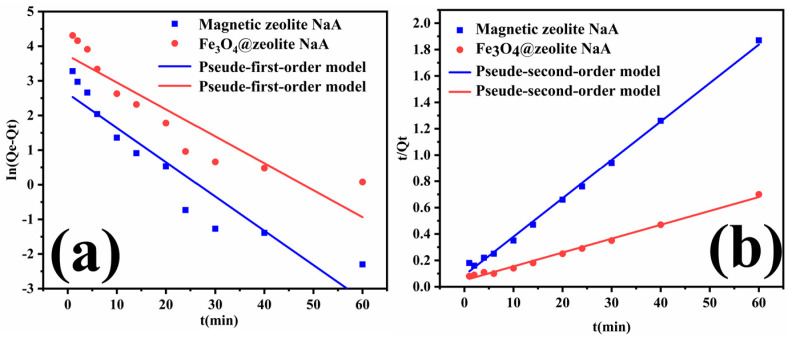
Pseudo-first order model (**a**) and pseudo-second order model (**b**) fittings for Cu^2+^ adsorption onto magnetic zeolite NaA and Fe_3_O_4_@zeolite NaA.

In order to further study the adsorption mechanism of magnetic zeolite NaA and Fe_3_O_4_@zeolite NaA for Cu^2+^. The adsorption kinetics of Cu^2+^ adsorption on magnetic zeolite NaA and Fe_3_O_4_@zeolite NaA were used for this research, which by the conditions of pH = 4, room temperature(298.15 K), initial concentration of 200 mg/L, adsorbent dose of 0.1 g, Cu^2+^ solution volume of 50 mL and different contact time. Two types of kinetics models are generally used and compared, namely the pseudo-first order and pseudo-second. The results and related parameters are shown in Figure 12 and Table 4, respectively. The results in Figure 12 and Table 4 show that the adsorption of Cu^2+^ on magnetic zeolite NaA and Fe_3_O_4_@zeolite NaA more followed the pseudo-second-order kinetic model (R^2^ = 0.99702, 0.99305), compared to the pseudo-first-order kinetic model (R^2^ = 0.89008, 0.84336), which implies that the adsorption process of Cu^2+^ by magnetic zeolite NaA and Fe_3_O_4_@zeolite NaA is of chemisorption mechanism [54]. 

In addition, the equilibrium adsorption capacity (Qe = 34.282, 95.147) of magnetic zeolite NaA and Fe_3_O_4_@zeolite NaA calculated by using the pseudo-second-order kinetic model is closer to the experimental data (Qe = 32.12, 86.54). The pseudo-second order rate parameters (K_2_) were higher for the magnetic zeolite NaA compared to those of the Fe_3_O_4_@zeolite NaA, which means magnetic zeolite NaA has a faster adsorption rate.

### 3.5. Adsorption Thermodynamics

**Figure 13:**
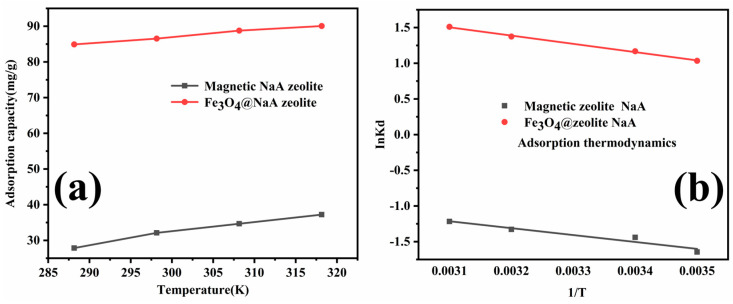
(**a**) Effect of temperature on adsorption of Cu^2+^ by magnetic zeolite NaA and Fe_3_O_4_@zeolite NaA (**b**).

The adsorption capacity of Cu^2+^ by magnetic zeolite NaA and Fe_3_O_4_@zeolite NaA at different temperatures was studied (Figure 13a). Adsorption thermodynamics of Cu^2+^ onto magnetic zeolite NaA and Fe_3_O_4_@zeolite NaA are studied, which under the conditions of initial concentration value of 200 mg/L, pH of 4, contact time of 24 min, adsorbent dose of 0.1 g, Cu^2+^ solution volume of 50 mL and temperature of 288.15 K, 298.15 K, 308.15 K and 318.15 K, respectively (Figure 13b).The relevant adsorption thermodynamic parameters are shown in Table 5. We found that the adsorption capcity of Cu^2+^ by magnetic zeolite NaA and Fe_3_O_4_@zeolite NaA increased with the increase of temperature (Figure 13a), which means that high temperature caused the increase of Cu^2+^ mobility [55]. However, the improvement of adsorption capacity is very small, which shows that different temperatures have little effect on the capacity of magnetic zeolite NaA and Fe_3_O_4_@zeolite NaA to adsorb Cu^2+^. The above results indicate that the magnetic zeolite NaA and Fe_3_O_4_@zeolite NaA at room temperature basically have the saturated adsorption capacity for Cu^2+^. It can be seen from Table 5 that the ΔG^o^ values of magnetic zeolite NaA and Fe_3_O_4_@zeolite NaA are both negative, and decrease with the increase of temperature, indicating that the adsorption process of magnetic zeolite NaA and Fe_3_O_4_@zeolite NaA to Cu^2+^ is spontaneous [56]. The values of ΔH^o^ are 8.05 and 9.64 KJ·mol^−1^, respectively, and both are between 20.9–418.4 KJ·mol^−1^, which proves once again that the adsorption of Cu^2+^ by magnetic zeolite NaA and Fe_3_O_4_@zeolite NaA is chemical adsorption and the adsorption is an endothermic process. [57]. The positive values of ΔS_0_ indicate that the adsorption of Cu^2+^ by magnetic zeolite NaA and Fe_3_O_4_@zeolite NaA is moving in the direction of increasing the chaos of the system [58,59,60].

## 4. Conclusions

A novel magnetic Cu^2+^ adsorbent Fe_3_O_4_@zeolite NaA was successfully prepared. XRD, FTIR, and TEM analyses showed that Fe_3_O_4_@zeolite NaA has good crystallinity and presents a typical core-shell structure. BET technical analysis showed that the specific surface area of Fe_3_O_4_@zeolite NaA reached 26.846 m^2^·g^−1^. VSM analysis shows that the saturation magnetization of Fe_3_O_4_@zeolite NaA is 5.38 emu/g, which means that it has the ability to quickly separate from the liquid phase under the action of an external magnetic field. The ability of Fe_3_O_4_@zeolite NaA to adsorb Cu^2+^ was tested, and it was found that the time to reach adsorption equilibrium was only 24 min. Under certain conditions (Adsorbent dose is 0.1 g; Cu^2+^ solution volume is 50 mL; Initial Cu^2+^ concentration is 200 mg/L^−1^; Temperature is 298.15 K; contact time is 24 min; pH = 4), Fe_3_O_4_@zeolite NaA has an adsorption capacity of 86.54 mg/g for Cu^2+^, and the Remove% value has reached 86.54%. The process of Fe_3_O_4_@zeolite NaA adsorption to Cu^2+^ has been studied by isotherm, kinetics and thermodyna. Adsorption data fit better with the Langmuir isotherm, suggesting that the adsorption is a monolayer adsorption. Kinetics study indicates that the adsorption follows the pseudo-second-order model, suggesting that is the chemical interaction between Cu^2+^ and adsorbent. The values of separation constant (0 < R_L_ <1) indicate that the adsorption for Cu^2+^ is a favorable process. Thermodynamic studies have shown that the adsorption of Fe_3_O_4_@zeolite NaA on Cu^2+^ is a spontaneous process with endothermic and entropy increase. In a word, Fe_3_O_4_@zeolite NaA is suitable as a Cu^2+^ adsorbent.

## Figures and Tables

**Table 1:** Textual properties of magnetic zeolite NaA and Fe_3_O_4_@zeolite NaA.

Sample	BET Surfac Area (m^2^·g^−1^)	Pore Volume (cm^3^·g^−1^)	Average Pore Size (nm)
Magnetic zeolite NaA	2.731	0.017872	31.7899
Fe_3_O_4_@zeolite NaA	26.846	0.136697	20.5846

**Table 2:** Isotherm constants for adsorption by magnetic zeolite NaA and Fe_3_O_4_@zeolite NaA.

Models	Parameter	Magnetic zeolite NaA	Fe_3_O_4_@zeolite NaA
	R^2^	0.93447	0.61082
Freundich	n	4.1406	4.5773
	K_F_ (mg/g)	9.9094	34.6927
	R^2^	0.99890	0.99734
Langmuir	Q_max_ (mg/g)	33.89	86.58
	K_L_ (L/mg)	0.10835	0.53177
	R^2^	0.96782	0.73113
Tempkin	B_1_	5.07216	9.8778
	K_t_ (L/mg)	3.85743	67.35653

**Table 3:** Comparison of different adsorbents for Cu^2+^ adsorption.

Adsorbent	Q_m_ (mg/g)	Ref.
Sewage sludge AC	7.73	[47]
Wheat shell	10.84	[50]
Lignocellulosic waste N-CDs	26.95	[51]
Mg2Al-LS-LD	71.4	[52]
Sulfonated MWCNT	36.8	[53]
Magnetic zeolite NaA	32.12	This study
Fe_3_O_4_@zeolite NaA	86.54	This study

**Table 4:** Kinetics constants for adsorption by magnetic zeolite NaA and Fe_3_O_4_@zeolite NaA.

Models	Parameter	Magnetic Zeolite NaA	Fe_3_O_4_@zeolite NaA
	K_1_ (min^−1^)	0.099	0.07775
Pseudo-first-order	Q_e_ (mg/g)	13.898	41.698
	R^2^	0.89008	0.84336
	K_2_ (min^−1^)	0.00969	0.00224
Pseudo-second-order	Q_e_ (mg/g)	34.282	95.147
	R^2^	0.99702	0.99305

**Table 5:** Thermodynamic parameters for Cu^2+^ adsorption on magnetic zeolite NaA and Fe_3_O_4_@zeolite NaA.

Sample	ΔH^o^	ΔS^o^	ΔG^o^(KJ·mol^−1^)			
	(KJ·mol^−1^)	[J·(K·mol)^−1^]	288.15 K	298.15 K	308.15 K	318.15 K
Magnetic zeolite NaA	8.05	14.86	−4.27	−4.42	−4.57	−4.72
Fe_3_O_4_@zeolite NaA	9.64	42.38	−12.19	−12.63	−13.05	−13.47

## Supplementary Materials

The following are available online at https://www.mdpi.com/1996-1944/13/21/5047/s1, Figure S1: TEM image of magnetic zeolite NaA, Figure S2: Separation ability test of Fe_3_O_4_@zeolite NaA, Table S1: XRF analyze of Fe_3_O_4_@SiO_2_ (w %).
