# Supplementary material for: Core-shell Fe3O4@zeolite NaA as an Adsorbent for Cu2+
Source: Materials (Basel). 2020 Nov 10;13(21):5047. doi: 10.3390/ma13215047 (PMC7665120; doi:10.3390/ma13215047)
Supplement: Supplementary file 1 [file materials-13-05047-s001.pdf]

materials

# Supplementary Materials

# Core-shell $Fe_3O_4$@zeolite NaA as an Adsorbent for $Cu^{2+}$

**Jun Cao [1], Peng Wang [2], Jie Shen [1] and Qi Sun [1,*]**

1 College of Materials and Metallurgy, Guizhou University, Guiyang 550025, China; juncaowyy@163.com (J.C.); cywandsyx@163.com (J.S.)
2 School of Optical and Electronic Information, Wuhan National Laboratory for Optoelectronics, Huazhong University of Science and Technology 1037 Luoyu Road, Wuhan 430074, China; pengwang180907@163.com

* Correspondence: qsun@gzu.edu.cn

Received: 7 October 2020; Accepted: 3 November 2020; Published: date

## SI Supporting Materials and Methods

*Batch Adsorption Experiments*

### S1.Preparation of $Cu^{2+}$ Solutions

Prepared a standard $Cu^{2+}$ stock solution (500 mg/L) by dissolving $Cu(SO_4)_2 \cdot 5H_2O$ in a beaker. The solution was then diluted to the desired concentration using deionized water.

### S2.Adsorption Study

All adsorption experiments were performed in a batch mode using a shaking table (MPS-1500, Ningbo, zhejiang province, China) at a controlled temperature. The pH of the solution was adjusted to the desired value by adding negligible volume of 0.1 M NaOH and 0.1 M HCl solution. The determination of pH value is measured by SMART PH818 PH meter. After the adsorption test, the sample was immediately filtered through a syringe filter (0.45 μm syringe filter, PP filter medida, Yuecheng, China), and the remaining $Cu^{2+}$ concentration in the supernatant was measured by inductively coupled plasma spectrometer (ICP- OES) analysis (8300, PerkinElmer, USA). The adsorption experiment was performed in a reciprocating water bath shaker at a shaking speed of 170rpm. The adsorption capacity (Qe, mg/g) and adsorption efficiency of the adsorbents were calculated using the following Equations (S1) and (S2) [1]:

$$Q_e = \frac{C_0 - C_e}{M} \times V \quad \textbf{(S1)}$$

$$R_{emoval\%} = \frac{C_0 - C_e}{C_0} \times 100 \quad \textbf{(S2)}$$

Where Qe is the adsorption capacity (mg/g); $C_0$ is the initial $Cu^{2+}$ concentration (mg/L); Ce is the final $Cu^{2+}$ concentration (mg/L); M (g) is the weight of the adsorbent used and V (L) is the volume of the $Cu^{2+}$ solution. $R_{emoval\%}$ is the removal rate of $Cu^{2+}$ by the adsorbent; Each experiment was repeated three times to obtain average results.

### Adsorption Kinetics Study

The knowledge of adsorption kinetics is important information for designing batch adsorption systems. To examine the adsorption kinetics of the adsorbent for $Cu^{2+}$, the Lagergren pseudo-first-order and pseudo-second-order kinetic models were examined [2].

(1) Lagergren pseudo-first-order kinetic model:

$$In(Q_e - Q_t) = In\,Q_e - K_1 t \quad \text{(S3)}$$

(2) Lagergren pseudo-second-order kinetic model:

$$\frac{t}{Q_t} = \frac{1}{K_2 Q_e^{\,2}} + \frac{t}{Q_e} \quad \text{(S4)}$$

where Qe is the amounts of adsorption at equilibrium, mg/g; $Q_t$ is the amounts of adsorption at time t, mg/g; $k_1$ is the first order rate constant, $min^{-1}$; $k_2$ is the second order rate constants, $min^{-1}$.

## Adsorption Isotherm Study

The liner form of Langmuir adsorption isotherm is one of the most famous well-adopted models used to describe the solid phase adsorption systems [3].

$$\frac{C_e}{Q_e} = \frac{C_e}{Q_{max}} + \frac{1}{K_L Q_{max}} \quad \text{(S5)}$$

$$R_L = \frac{1}{1 + K_L C_0} \quad \text{(S6)}$$

Where $Q_{max}$ is the Langmuir maximum adsorption capacity (mg/g), and $K_L$ is the Langmuir binding constant. Where $C_0$ and $K_L$ are the initial concentration of arsenic and Langmuir isotherm constant. If the value of $O < R_L < 1$, it represents favourable adsorption.

The Freundlich model indicates the heterogeneity of the adsorbent surface and considers multilayer adsorption. The linear form of Freundlich adsorption model is as follows [4]:

$$InQ_e = Ink_f + \frac{1}{n} InC_e \quad \text{(S7)}$$

Where $K_f$ and 1/n are Freundlich constants, related to adsorption capacity and adsorption intensity (heterogeneity factor) respectively. The values of $K_f$ and 1/n were obtained from the slope and intercept of the linear Freundlich plot of ln $Q_e$ versus ln $C_e$.

The Temkin model is a modification equation of the Langmuir model. The adsorption enthalpy declined linearly with the increase of adsorption amounts. The Temkin model is described by equation S8:

$$Q_e = B_1 InK_t + B_1 InC_e \quad \text{(S8)}$$

where $B_1$ and $K_t$ are constants related to adsorption enthalpy and capacity of the adsorbent.

## Thermodynamic Study

Using the slope and intercept of the plot the $\ln k_d$ versus 1/T is presented in Equation 9, the enthalpy change (ΔH°) and entropy change (ΔS°) can be calculated. T(K) is the Kelvin temperature. R(8.314J/(mol·K)) is the universal gas constant. The Gibbs free-energy change of the sorption of $Cu^{2+}$ ions using adsorbent is given using Equation 9:

$$K_d = \frac{Q_e}{C_e}$$

$$InK_d = \frac{\Delta S^0}{R} - \frac{\Delta H^0}{RT} \quad \textbf{(S9)}$$

The standard enthalpy change (ΔH°), and standard entropy change (ΔS°) was calculated using following equation [5]:

$$\Delta G^0 = \Delta H^0 - T\Delta S^0 \quad \textbf{(S10)}$$

Where ($\Delta H^0$) is standard enthalpy change (KJ/mol) and ($\Delta S^0$) is standard entropy change (KJ/mol K).

**Table S1.** XRF analyze of $Fe_3O_4$@$SiO_2$ (w %).

| **Items** | **Content(w %)** |
|---|---|
| $Fe_3O_4$ | 47.003 |
| $SiO_2$ | 51.663 |

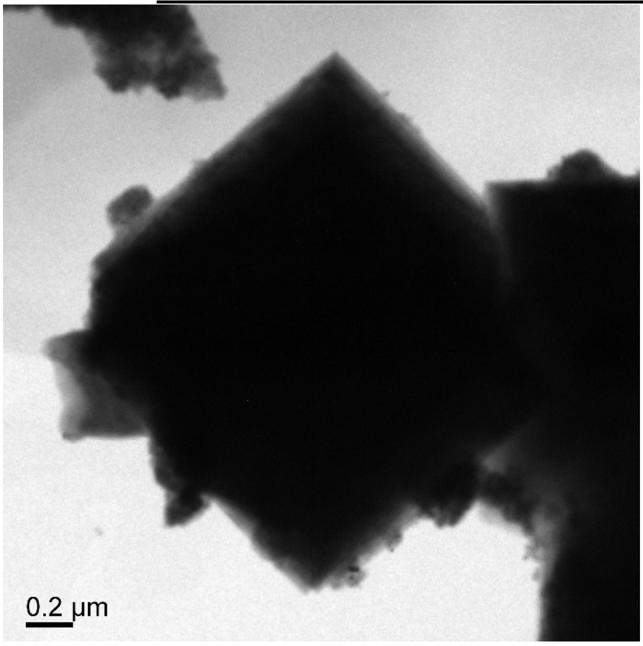


**Figure S1.** TEM image of magnetic zeolite NaA.

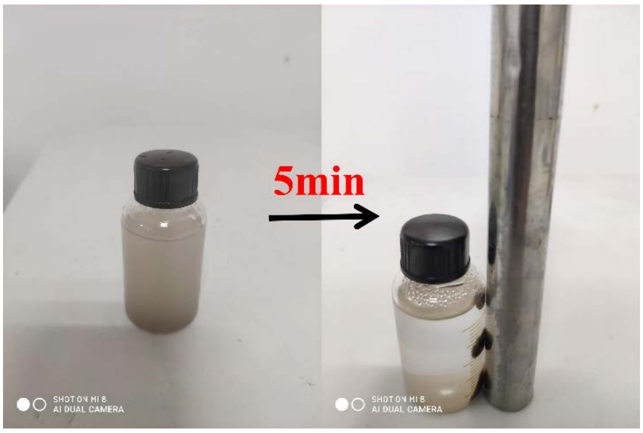


**Figure S2.** Separation ability test of $Fe_3O_4$@zeolite NaA.

## References

1. Choi J W, Kim H J, Ryu H, et al. Three-dimensional double-network hydrogels of graphene oxide, alginate, and polyacrylonitrile for copper removal from aqueous solution. Environmental Engineering Research 2019; 25(6): 923-928.
2. K K H Choy, G McKay, Sorption of cadmium, copper, and zinc ions onto bone char using Crank diffusion model. Chemosphere 2005; 60: 1141–1150.
3. Abd El-Magied, M O Galhoum, A A Atia, A A Tolba, A A Vincent, T.; Guibal, E Maize M S. Cellulose and Chitosan Derivatives for Enhanced Sorption of Erbium(III). Colloids Surf. A: Physicochem. Eng. Aspects 2017; 529: 580-593. .
4. Pillewan P, Mukherjee S, Roychowdhury T, Das S, Bansiwal A, Rayalu, S. Removal of As(III) and As(V) from water by copper oxide incorporated mesoporous alumina. Journal of Hazardous Material 2011; 186: 367-375.
5. Sekar M, Sakthi V, Rengaraj, S. Kinetics and equilibrium adsorption study of lead(II) onto activated carbon prepared from coconut shell. Journal of Colloid Interface Science 2004; 279: 307-313.

© 2020 by the authors. Licensee MDPI, Basel, Switzerland. This article is an open access article distributed under the terms and conditions of the Creative Commons Attribution (CC BY) license (http://creativecommons.org/licenses/by/4.0/).
